# A Gα12-specific Binding Domain in AKAP-Lbc and p114RhoGEF

**DOI:** 10.5334/1750-2187-11-3

**Published:** 2016-09-09

**Authors:** Joseph W. Martin, Kyle S. Cavagnini, Douglas N. Brawley, Carrie Y. Berkley, William C. Smolski, Ricardo D. Garcia, Autumn L. Towne, Jonathan R. Sims, Thomas E. Meigs

**Affiliations:** Department of Biology, University of North Carolina Asheville, One University Heights, Asheville NC 28804, USA

**Keywords:** Gα12, Gα13, heterotrimeric G protein, AKAP-Lbc, p114RhoGEF, Rho

## Abstract

AKAP-Lbc is a Rho-activating guanine nucleotide exchange factor (RhoGEF) important in heart development and pro-fibrotic signaling in cardiomyocytes. Heterotrimeric G proteins of the G12/13 subfamily, comprising Gα12 and Gα13, are well characterized as stimulating a specialized group of RhoGEFs through interaction with their RGS-homology (RH) domain. Despite lacking an RH domain, AKAP-Lbc is bound by Gα12 through an unknown mechanism to activate Rho signaling. We identified a Gα12-binding region near the C-terminus of AKAP-Lbc, closely homologous to a region of p114RhoGEF that we also discovered to interact with Gα12. This binding mechanism is distinct from the well-studied interface between RH-RhoGEFs and G12/13 α subunits, as demonstrated by Gα12 mutants selectively impaired in binding either this AKAP-Lbc/p114RhoGEF region or RH-RhoGEFs. AKAP-Lbc and p114RhoGEF showed high specificity for binding Gα12 in comparison to Gα13, and experiments using chimeric G12/13 α subunits mapped determinants of this selectivity to the N-terminal region of Gα12. In cultured cells expressing constitutively GDP-bound Gα12 or Gα13, the Gα12 construct was more potent in exerting a dominant-negative effect on serum-mediated signaling to p114RhoGEF, demonstrating coupling of these signaling proteins in a cellular pathway. In addition, charge-reversal of conserved residues in AKAP-Lbc and p114RhoGEF disrupted Gα12 binding for both proteins, suggesting they harbor a common structural mechanism for interaction with this α subunit. Our results provide the first evidence of p114RhoGEF as a Gα12 signaling effector, and define a novel region conserved between AKAP-Lbc and p114RhoGEF that allows Gα12 signaling input to these non-RH RhoGEFs.

## Introduction

Cells respond to changes in their environment through complex networks of intracellular signaling proteins. Many responses are initiated at the cell surface by external stimuli that bind seven-transmembrane-span receptors, which are physically coupled to heterotrimeric guanine nucleotide binding proteins (G proteins) on the cytoplasmic face of the plasma membrane. Receptor activation causes the α subunit of the G protein to release GDP and bind GTP, triggering heterotrimer dissociation into two signaling entities: the activated, GTP-bound α subunit and a stable dimer of β and γ subunits. Activated α subunits bind and stimulate a variety of downstream effectors that include kinases, phosphatases, generators of second messengers, ion channels, and transcriptional regulators. Based on amino acid sequence comparison, G protein α subunits are grouped into four subfamilies: Gs, Gi, Gq, and G12/13. The G12/13 subfamily, comprising Gα12 and Gα13 in mammals, has been implicated in pathways that regulate cell polarity, proliferation, migration and invasion, cytoskeletal rearrangements, angiogenesis during embryonic development, and other cellular pathways and physiologic events [1]. Many G12/13 mediated responses require downstream activation of Rho, a small GTPase that, when activated by exchange of bound GDP for GTP, drives signaling through effector proteins that include mDia and Rho-associated kinase [2]. The classical link between the G12/13 subfamily and Rho is a small subclass of Rho-directed guanine nucleotide exchange factors (RhoGEFs) comprising p115RhoGEF, leukemia-associated RhoGEF (LARG), and PDZ-RhoGEF. These proteins are bound directly by GTP-bound α subunits of the G12/13 subfamily, triggering the RhoGEF to bind and activate Rho [3]. A common feature of these G12/13-responsive RhoGEFs is an RGS-homology (RH) domain, similar in sequence to a hallmark structural region in regulators of G protein signaling (RGS) that bind activated α subunits and accelerate their GTP hydrolysis and consequent inactivation [4]. The structural determinants of Gα-RhoGEF interaction have been studied in fine detail, most prominently via crystallographic analyses of Gα13 bound to a region of p115RhoGEF and PDZ-RhoGEF encompassing the RH domain [5,6,7].

Despite rapid advances in our understanding of G12/13 signaling through RH-RhoGEFs, it is increasingly evident that other, less-understood signaling pathways utilize this G protein subfamily. Despite sharing 67% amino acid identity, Gα12 and Gα13 have diverse, non-overlapping sets of binding partners and a variety of distinct signaling roles [8,9]. One protein identified as a downstream binding partner of Gα12 is AKAP-Lbc, a member of the family of A-kinase anchoring proteins (AKAP) that harbor a common function of binding cyclic AMP-dependent protein kinase and directing its subcellular targeting [10]. AKAP-Lbc is a specialized AKAP of 2817 amino acids, harboring a 893-residue region identical to the RhoGEF proto-Lbc. Initial studies of AKAP-Lbc revealed its interaction with Gα12 as a mechanism for stimulating RhoA activation and assembly of actin stress fibers [11,12]. AKAP-Lbc expression appears highest in the heart among tissues examined, whereas Gα12 is expressed in the heart and numerous other cell types [13]. A mouse knockout model showed AKAP-Lbc as playing an essential role in embryonic heart development by mediating Gα12 signaling [14]. In addition, Gα12 stimulation of AKAP-Lbc appears to be an important mechanism in cardiac disease states such as cardiomyocyte hypertrophy mediated by the α1-adrenergic receptor [15,16] and pro-fibrotic signaling in which type 1 angiotensin II receptors drive cardiac myocytes to differentiate into matrix-secreting myofibroblasts [17].

Although the Gα12-AKAP-Lbc-RhoA signaling axis is implicated in several physiologic and pathologic events, the structural determinants in Gα12 and AKAP-Lbc that facilitate their interaction and subsequent activation of Rho are not known. AKAP-Lbc and the RH-RhoGEFs have functional similarity in providing a conduit between the G12/13 subfamily and Rho, and share characteristic features of Rho-directed GEFs: a Dbl-homology (DH) domain that catalyzes nucleotide exchange on Rho and an adjacent pleckstrin-homology (PH) domain [18]. However, AKAP-Lbc lacks an RH domain [3,12]. In p115RhoGEF, LARG, and PDZ-RhoGEF, the RH domain provides a crucial surface for G12/13 α subunits to bind the protein and stimulate its RhoGEF activity [19,20,21]. Crystallographic analysis of Gα13 in complex with the isolated RH domain of p115RhoGEF revealed residues in both proteins crucial for this interaction [7]. Also, ectopic expression of isolated RH domains exerted dominant-negative effects on Gα12 and Gα13 signaling toward cell growth and tumorigenic responses [15,20,22,23,24]. The absence of an RH domain in AKAP-Lbc suggests the G12/13 subfamily utilizes a different, unknown mechanism for binding this protein and stimulating its activity toward Rho, in comparison to RH-RhoGEFs.

In this study, we identify a region of AKAP-Lbc/proto-Lbc that interacts with Gα12, and demonstrate that another RhoGEF lacking an RH domain, p114RhoGEF, harbors a homologous region that also binds Gα12. We further show that these regions of AKAP-Lbc and p114RhoGEF bind exclusively to Gα12 within the G12/13 subfamily, in contrast to RH-RhoGEFs that exhibit bias toward Gα13. In addition, we identify amino acids common to AKAP-Lbc and p114RhoGEF that are involved in Gα12 interaction, suggesting Gα12-specific binding evolved in a common ancestor of this region that has remained similar in these two RhoGEFs. Reciprocally, we identify a point mutant of Gα12 disrupted in binding AKAP-Lbc and p114RhoGEF but not LARG, suggesting Gα12 harbors structural features for interaction with AKAP-Lbc and p114RhoGEF that are distinct from its RH-RhoGEF binding mechanism. Finally, utilizing cultured cells, we show that constitutively inactive Gα12 exerts a dominant-negative effect on serum-dependent growth signaling through p114RhoGEF, and that the Gα12-binding region of p114RhoGEF disrupts signaling by activated Gα12 but not Gα13.

## Methods

***DNA constructs.*** A plasmid harboring the AKAP-Lbc cDNA was provided by Dario Diviani (Univ. of Lausanne, Switzerland), the plasmid encoding myc-tagged p114RhoGEF was a gift from Tatyana Voyno-Yasenetskaya (University of Illinois at Chicago), and SRE-luciferase was provided by Channing Der (Univ. of North Carolina, Chapel Hill). Gα12/Gα13 chimeras were provided by Barry Kreutz (University of Illinois at Chicago). GST-fusion constructs encoding 257-residue and 106-residue regions of AKAP-Lbc and p114RhoGEF were generated by PCR amplification from these cDNAs, with amplimers ligated into pGEX-KG. GST fusions of the RH domains of p115RhoGEF and LARG are described previously [25]. All Gα12 and Gα13 constructs used in our study were housed in the plasmid pcDNA3.1 (Invitrogen). Internally myc-tagged Gα12 in its constitutively active (Q229L) or constitutively GDP-bound (G228A) form was engineered as described previously [25]. For engineering an internal myc tag in constitutively active (Q226L) and constitutively GDP-bound (G225A) Gα13, we first used the Quik-Change II kit (Agilent Technologies, Santa Clara, CA) to replace Met-136 with a Pro residue, creating a Pro-Val motif in which the codons harbored an AgeI restriction site. Next, we excised the myc coding sequence from tagged Gα12 using AgeI and ligated this into Gα13. To generate EGFP-tagged Gα12 and Gα13 constructs, EGFP coding sequence was PCR-amplified from the plasmid pEGFP-C1 with AgeI sites incorporated upstream and downstream of codons for the start Met and C-terminal Lys, and this amplimer was ligated into Gα12 and Gα13 plasmids in place of their excised myc tags. Gα12/Gα13 chimeras (gift of Barry Kreutz, Univ. of Illinois at Chicago) were subject to PCR amplification and each coding sequence ligated into pcDNA3.1 (Invitrogen), to provide a host plasmid lacking an AgeI site. These chimeras were tagged internally with EGFP at an Agel site we engineered using the approach described above. For each Gα12 and Gα13 construct, the myc or EGFP sequence was flanked upstream and downstream by flexible linkers composed of the sextet Ser-Gly-Gly-Gly-Gly-Ser [25,26]. In summary, all tagged Gα12 and Gα13 constructs and their chimeras were confirmed by sequencing to harbor the sequence Ser-Gly-Gly-Gly-Gly-Ser-[myc tag or EGFP]- Ser-Gly-Gly-Gly-Gly-Ser, flanked upstream and downstream by a Pro-Val motif in the helical domain.

***Expression and immobilization of proteins.*** GST-fusion constructs were transformed into BL21(Gold)-DE3 cells (Agilent Technologies) and single colonies used to inoculate 5-mL liquid cultures for growth to saturation at 37°C under 75 µg/mL ampicillin selection. These cultures were placed in 150-mL cultures under the same conditions and grown to OD_600_ of 0.5–0.7, and then recombinant protein expression was induced for 3 h using 0.5 mM isopropyl-*β*-D-thiogalactopyranoside. Cells were lysed on ice using 0.32 mg/mL lysozyme (MP Biomedicals), and GST fusion proteins were bound to Glutathione Sepharose 4B (GE Healthcare) pre-washed in binding buffer (50 mM Tris pH 7.7, 1 mM EDTA, 1 mM dithiothreitol). High-speed supernatant from lysed cells was combined with Glutathione Sepharose and mixed by inversion at 4°C. After three cycles of pelleting beads at 1300 *g* and washing with binding buffer supplemented with 150 mM NaCl, suspensions of Sepharose beads with captured proteins were snap-frozen in aliquots and stored at –80°C.

***Protein interaction assays.*** Human embryonic kidney cells (HEK293) were grown in Dulbecco’s modification of Eagle’s medium (Corning, Tewksbury MA) supplemented with 10% fetal bovine serum (Quality Biological, Gaithersburg MD). For myc- and EGFP-tagged Gα12 and Gα13 variants, 7.0 µg of plasmid DNA was transfected into a 10-cm dish of HEK293 cells at approximate 90% confluence, using Lipofectamine 2000 (Invitrogen) in accordance with the manufacturer’s instructions. After 42–48 h, dishes of cells were placed on ice, washed with 10 mL cold PBS, scraped in 3 mL cold PBS, and centrifuged 5 min at 500g, 4°C. Cell pellets were resuspended in Lysis Buffer (50 mM HEPES pH 7.5, 1 mM EDTA, 3 mM dithiothreitol, 10 mM MgSO_4_, 1% (w/v) polyoxyethylene-10-lauryl ether) containing the protease inhibitors leupeptin (2 µM), pepstatin (1.5 µM), 4-(aminoethyl)benzenesulfonyl fluoride hydrochloride (1.7 mM), TLCK (58 µM), TPCK (61 µM), and phenylmethylsulfonyl fluoride (267 µM), and then inverted 30 min at 4°C and centrifuged at 80,000 *g* for 1 h. These high-speed supernatants were diluted in Lysis Buffer lacking polyoxyethylene-10-lauryl ether to dilute this detergent to 0.05% (w/v), and 5% of this volume was set aside on ice for each sample as “load”. The remainder of diluted high-speed supernatant was combined with Sepharose-bound GST fusion proteins and incubated 2 h at 4°C with continuous inversion. Samples were centrifuged 3 min at 1,300 *g*, 4°C, and pellets were washed three times in Lysis Buffer containing 0.05% (w/v) polyoxyethylene-10-lauryl ether and then denatured in Laemmli sample buffer for 10 min at 72°C. Precipitates and loads were subjected to SDS-PAGE and immunoblot analysis using antibodies specific to the Gα12 N-terminus (Santa Cruz Biotechnology), full-length Gα13 (clone 6F6-B5, EMD Millipore, Billerica MA), myc epitope tag (EMD Millipore), or EGFP (Thermo Scientific), followed by anti-rabbit or anti-mouse alkaline phosphatase-conjugated secondary antibodies (Promega). Immunoblots were developed using NBT/BCIP colorimetric detection in alkaline phosphatase buffer, and imaged using a Kodak Gel Logic 100 system.

***SRE-mediated transcriptional activation assays.*** HEK293 cells grown in 12-well plates were transfected with 0.2 µg SRE-luciferase plasmid (provided by Channing Der, UNC-Chapel Hill) and 0.02 µg of pRL-TK that contains the cDNA for *Renilla* luciferase, plus co-transfected plasmids encoding myc-tagged p114RhoGEF or myc- or EGFP-tagged variants of Gα12 or Gα13. Assays for SRE-mediated transcriptional activation were performed as described previously [27]. Briefly, cells were washed with phosphate-buffered saline and lysed in passive lysis buffer (Promega). Lysates were cleared 1 min at 16000 *g* and then 25% of each supernatant was denatured in Laemmli sample buffer for 10 min at 72°C for immunoblot analysis. Remaining supernatants were analyzed for SRE-driven firefly luciferase expression and an internal control of *Renilla* luciferase expression using a dual-luciferase assay system and GloMax 20/20 luminometer (Promega). For each sample, light output from SRE-driven luciferase activity was divided by light output from pRL-TK luciferase activity to normalize for variations in transfection efficiency.

## Results

**Figure 1:**
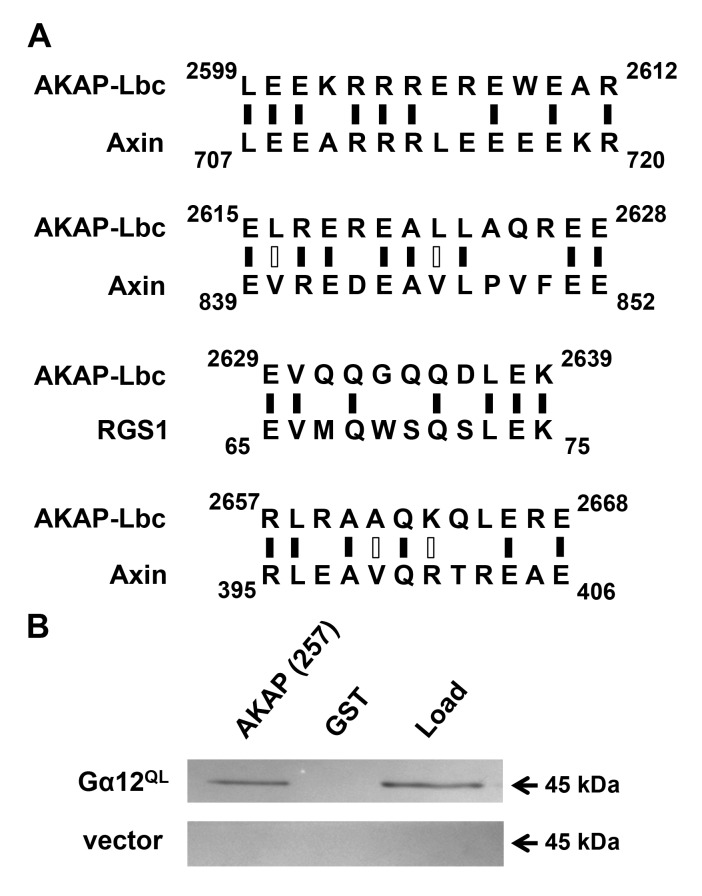
Identification of a Gα12-interacting region in AKAP-Lbc. **(A)** Sequence alignment of AKAP-Lbc with other Gα12 signaling targets. Results of Expasy SIM alignment using amino acid sequences of human proteins AKAP-Lbc (GenBank: NP_006729), axin-1 (NP_003493), and RGS1 (AAH15510) are shown. Regions of AKAP-Lbc excluding the tandem DH/PH domains were examined for homology to short sequences within RGS-1 and axin. Black vertical dashes indicate identical residues, open dashes indicate residues with similar properties. **(B)** Binding of Gα12 to an AKAP-Lbc region similar to axin and RGS1. HEK293 cells were transfected with a plasmid encoding myc-tagged, constitutively active Gα12 (GTPase-deficient Q229L mutant), or with pcDNA3.1 vector, and detergent-soluble extracts prepared as described in Methods. For each transfected sample, 5% of diluted extract was set aside as starting material (*Load*), and co-precipitation assays were performed using a Sepharose-bound GST-fusion of a 257-residue region of AKAP-Lbc, or GST alone. After SDS-PAGE and electroblot transfer, nitrocellulose membranes were probed with anti-Gα12 antibody (Santa Cruz Biotechnology; sc-409). Immunoblot images shown are representative of >5 experiments, with bands visible at the expected size (~45 kDa) for myc-tagged Gα12 and not detected in vector-transfected samples.

**Identification of a Gα12-interacting region in AKAP-Lbc.** AKAP-Lbc was discovered by Diviani, Scott and colleagues as a RhoGEF stimulated by Gα12 binding to drive Rho activation [12]. Gα12 also was shown to interact with proto-Lbc, a shorter splice variant encoded by the AKAP-Lbc gene but lacking the N-terminal A-kinase anchoring domain [28,29]. The mechanism of functional interaction between Gα12 and AKAP-Lbc is unknown. To identify determinants of Gα12 binding within AKAP-Lbc, we aligned its sequence with other Gα12 target proteins. Gα12 was reported as more potent than Gα13 in stimulating RhoGEF activity of AKAP-Lbc [12], and therefore we selected RGS1 and axin for these alignments due to their preferential binding of Gα12 within the G12/13 subfamily [30,31]. Although axin and RGS1 harbor a core “RGS box” [32], we predicted any Gα12-specific binding motifs shared between these proteins and AKAP-Lbc would reside outside this RGS box, due to absence of RGS-homologous sequence in AKAP-Lbc [3]. As shown in Figure 1A, several short sequences near the AKAP-Lbc C-terminus exhibit homology either to axin or a RGS1 motif that resides adjacent to its RGS box. Based on these findings, we isolated the C-terminal 257-amino acid region of AKAP-Lbc encompassing these motifs, including 35 residues upstream of the N-terminal-most motif to improve its chances of correct folding. This polypeptide was expressed in *E. coli* as a fusion to glutathione-S-transferase (GST) and subsequently purified with glutathione-coated Sepharose. In co-precipitation experiments, this AKAP-Lbc region interacted robustly with constitutively active (Q229L) Gα12 from lysates of transiently transfected HEK293 cells (Figure 1B). These results indicate the C-terminal 257 residues of AKAP-Lbc harbor key determinants of Gα12 interaction.

**Figure 2:**
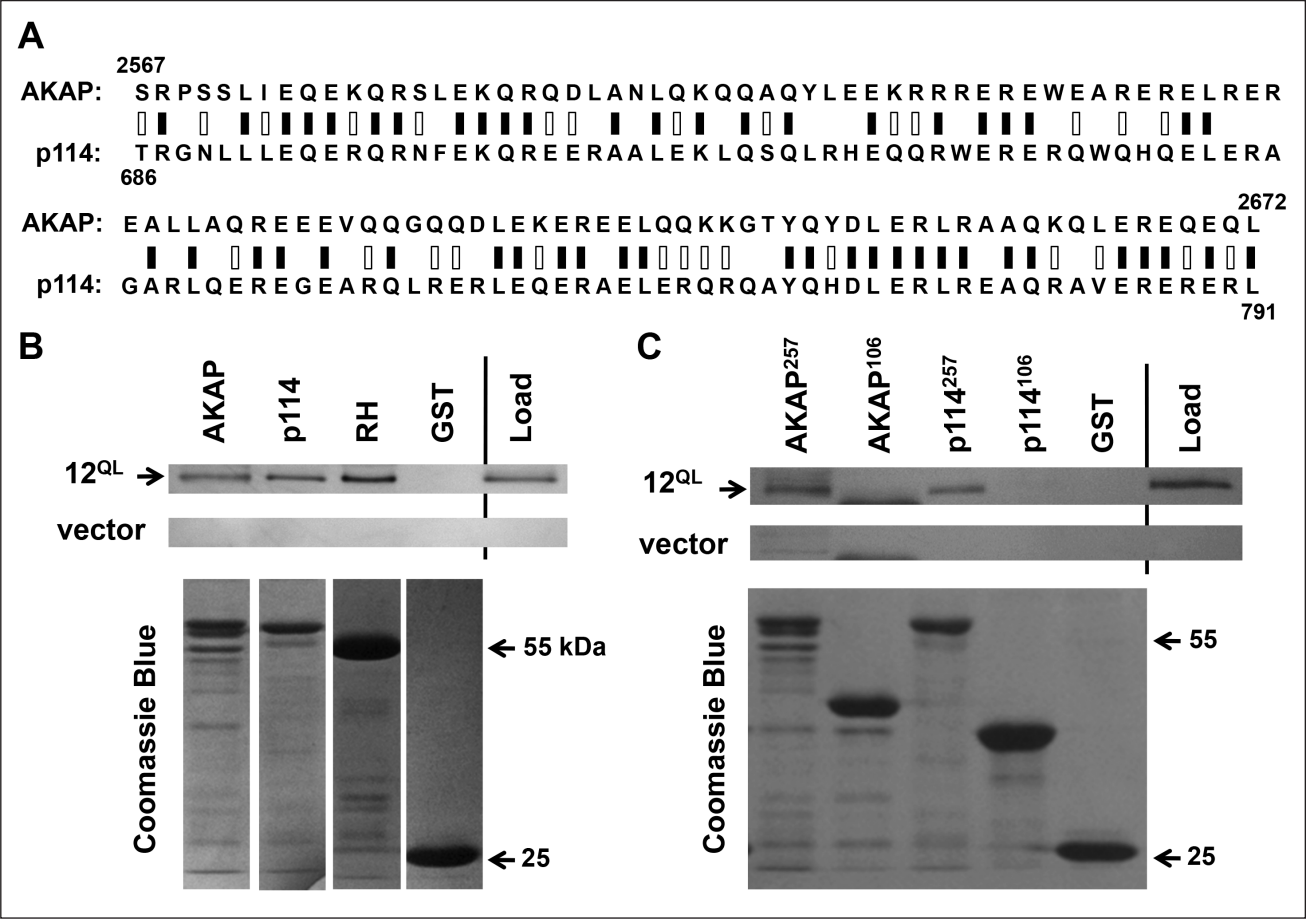
A conserved Gα12-binding domain in AKAP-Lbc and p114RhoGEF. **(A)** Sequence alignment of AKAP-Lbc and p114RhoGEF. Results of Protein BLAST (blastp) analysis are displayed, with black bars indicating identical residues (50/106), white bars indicating non-identical positive matches (28 additional residues), and zero gaps. **(B)** Interaction of Gα12 with regions of p114RhoGEF and AKAP-Lbc. Co-precipitation experiments were performed as described in Methods, using GST-fusions of the 257-amino acid C-terminus of AKAP-Lbc (*AKAP*), the corresponding 257 residues of p114RhoGEF (*p114*), amino acids 2-252 of p115RhoGEF containing its RH domain (*RH*), and GST alone. Load samples were set aside prior to addition of Sepharose-bound GST-fusion proteins. Co-precipitations from HEK293 cells transfected with myc-tagged, constitutively activated Gα12 (*12*^QL^) and empty vector were performed in parallel. For each sample, 20% of precipitated material was examined by SDS-PAGE/Coomassie Blue staining to assess levels of GST-fusion proteins, shown in lower panels. **(C)** Co-precipitations using the closely homologous, 106-amino acid domains within the Gα12-binding, 257-residue regions of AKAP-Lbc (*AKAP*) and p114RhoGEF (*p114*) are shown. Amino acid lengths of these adducts to GST are indicated as superscripts. Results in **(B)** and **(C)** are representative of three or more independent experiments.

**A conserved Gα12-binding domain in AKAP-Lbc and p114RhoGEF.** We examined this 257-amino acid region of AKAP-Lbc for homology to other human proteins using the Basic Local Alignment Search Tool (BLAST; National Library of Medicine). The protein with closest match to this region was p114RhoGEF (ArhGEF18), which harbors 47% identity with AKAP-Lbc in a 106-residue subregion (Figure 2A). This region of close homology spans amino acids 2567-2672 in AKAP-Lbc and 686-791 in p114RhoGEF. An interesting aspect of this finding was that p114RhoGEF, like AKAP-Lbc, lacks an RH domain [33]. We hypothesized these sequences in AKAP-Lbc and p114RhoGEF might define a common Gα12-interacting domain, and therefore engineered a GST-fusion of this p114RhoGEF region. Similar to our AKAP-Lbc construct, this p114RhoGEF sequence contained the core 106 residues flanked by native amino acids upstream and downstream to produce a 257-amino acid polypeptide. This region of p114RhoGEF bound constitutively active Gα12 under the same conditions in which Gα12 interacted with AKAP-Lbc, with band intensity comparable to co-precipitation by the RH domain of p115RhoGEF (Figure 2B). These results provide the first evidence of interaction between Gα12 and p114RhoGEF, and further suggest the region of homology between AKAP-Lbc and p114RhoGEF defines a novel binding surface for Gα12.

Close homology between these Gα12-interacting regions of AKAP-Lbc and p114RhoGEF is limited to the 106-residue span indicated in Figure 2A; sequences in our 257-amino acid constructs upstream and downstream of this “core” sequence show essentially no similarity. Therefore, we tested whether these 106 amino acids expressed in isolation are sufficient for interaction with Gα12. A GST-fusion of this sequence from AKAP-Lbc or p114RhoGEF was unable to co-precipitate constitutively active Gα12 (Figure 2C). These results suggest the protein regions flanking this core subregion provide necessary context for correct folding and Gα12 binding when expressed as a GST-fused polypeptide.

**Figure 3:**
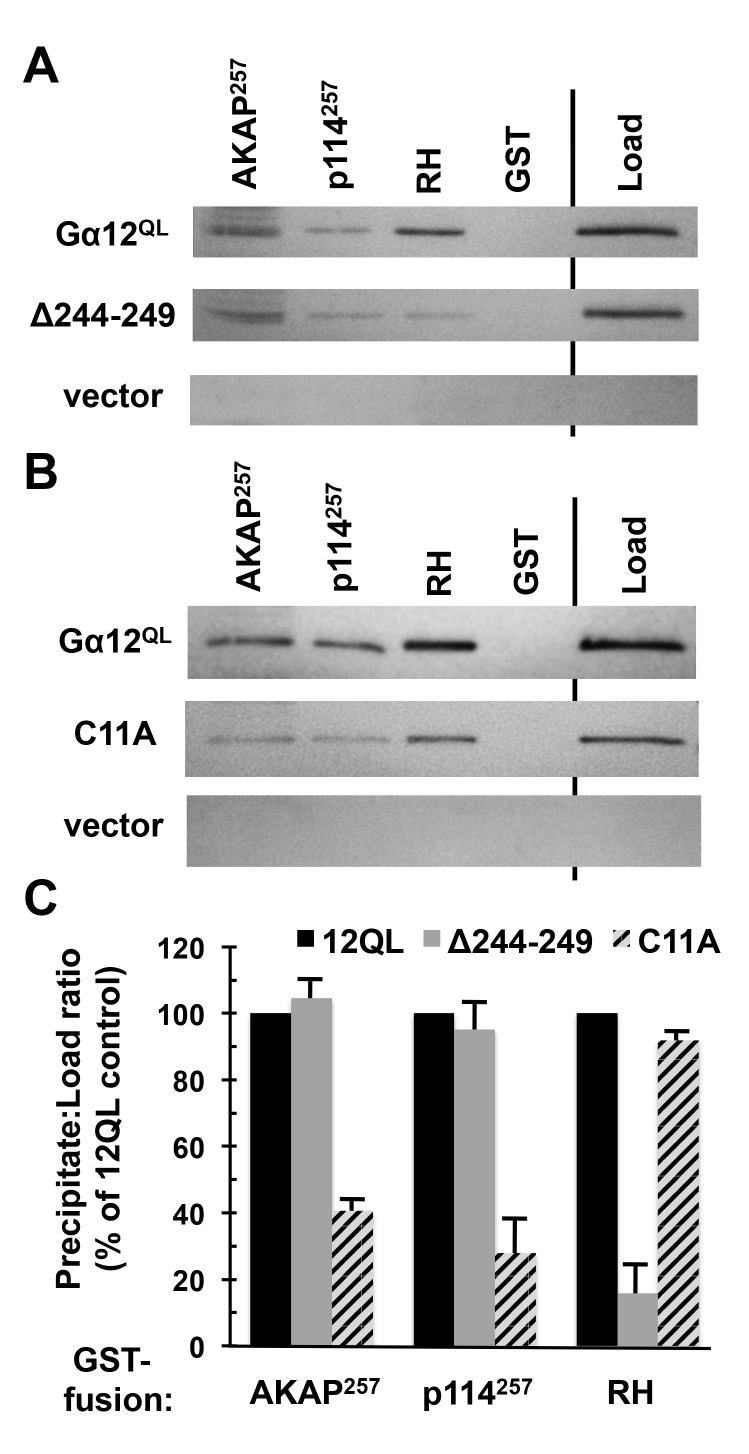
Gα12 binding to AKAP-Lbc and p114RhoGEF is distinct from RH-RhoGEF interaction. **(A, B)** Results of protein interaction experiments using mutant forms of myc-tagged, constitutively active Gα12 (Gα12^QL^). For both panels, Sepharose-immobilized AKAP-Lbc and p114RhoGEF constructs utilized in Figure 2B were tested, alongside the immobilized RH domain of LARG (*RH*), for ability to co-precipitate Gα12 variants from HEK293 cell extracts as described in Methods. Load samples were set aside prior to the precipitation step. Representative results are shown. **(C)** Immunoblot bands for Gα12 mutants were quantified using ImageJ, and for each sample the precipitate:load ratio was calculated and presented as a % of the precipitate:load ratio for non-mutated, constitutively active Gα12. Results shown are from three or more independent experiments, with graphs indicating mean ± standard error of the mean (s.e.m.).

**Gα12 binding to AKAP-Lbc and p114RhoGEF is distinct from RH-RhoGEF interaction.** The structural determinants that allow the G12/13 subfamily to engage RH-RhoGEFs are well-studied, most notably by crystallographic analysis of Gα13 in complex with isolated RH domains [6,7], and several mutations in the α subunit are disruptive to this interaction [34,35,36]. To determine whether the mechanism of Gα12 interaction with AKAP-Lbc and p114RhoGEF is distinct from canonical RH-RhoGEF binding, we examined a Gα12 cassette mutant previously characterized as impaired in binding the RH domains of LARG and p115RhoGEF while retaining signaling through other, non-Rho-mediated pathways. This mutant (Δ244–249) contains the sextet Asn-Ala-Ala-Ile-Arg-Ser in place of native Gα12 sequence between the switch II and III regions [35]. To measure levels of this Gα12 cassette mutant and its non-substituted control, it was important to have a means for detecting these recombinant Gα12 variants on immunoblots without interference from endogenous Gα12. Therefore, a myc epitope tag was positioned in constitutively active Gα12 and its Δ244–249 mutant within the αB–αC loop of the helical domain, a location found previously to tolerate tag insertion in Gαq and Gα12 without disrupting effector binding or downstream signaling by the α subunit [25,26,27]. In co-precipitation experiments, the Δ244–249 substitution caused no disruption of constitutively active Gα12 binding to AKAP-Lbc and p114RhoGEF regions (Figure 3A, C). In contrast, binding of this mutant to the LARG RH domain was greatly reduced in comparison to normal, constitutively active Gα12, consistent with previous results [35].

To further test the hypothesis that Gα12 interaction with AKAP-Lbc and p114RhoGEF utilizes a mechanism distinct from RH-RhoGEF binding, we sought Gα12 amino acids demonstrated as important in Rho-dependent tumorigenic signaling but located in regions of the α subunit not implicated in RH-RhoGEF interaction. One such amino acid is Cys-11, which provides a site for post-translational palmitoylation of Gα12 and is required for the activated protein to drive a transformed growth state in cultured fibroblasts [37]. Because cassette substitution of the amino acid sextet encompassing this Cys residue did not impair interaction with LARG in our previous work [25], we inferred this Cys does not participate in RH-RhoGEF binding. Therefore, we engineered a C11A substitution in myc-tagged, constitutively active Gα12 and found this mutation to hinder interaction with the 257-residue AKAP-Lbc construct (~60% loss of binding as calculated by co-precipitated Gα12 normalized for starting material) and the corresponding p114RhoGEF construct (~72% loss of binding). Conversely, C11A substitution caused no loss of binding to the RH domain of LARG (Figure 3B, C), suggesting lipid modification of the α subunit at this Cys residue is uniquely required for interaction with the non-RH RhoGEFs AKAP-Lbc and p114RhoGEF.

**Figure 4:**
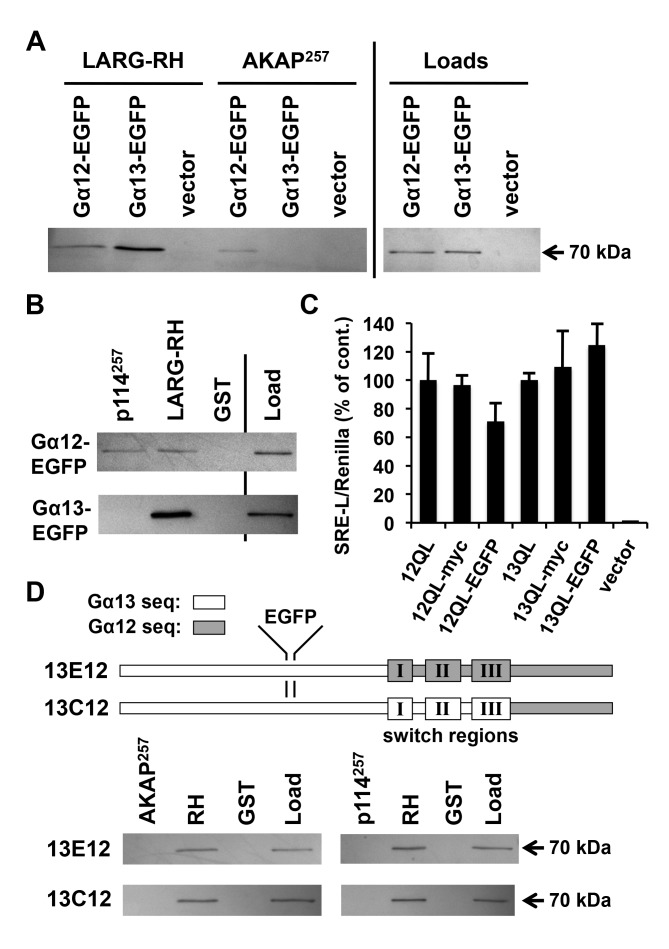
AKAP-Lbc and p114RhoGEF are selective for Gα12 binding. **(A, B)** Binding of EGFP-tagged G12/13 α subunits to RhoGEF domains. GST-fusions of the 257-amino acid AKAP-Lbc and p114RhoGEF regions used in Figure 2B, as well as the RH domain of LARG, were examined for ability to co-precipitate EGFP-tagged, constitutively active Gα12 and Gα13. Unmodified GST was tested as a negative control for α subunit co-precipitation. Shown are immunoblot analyses of precipitates and loads using an anti-EGFP antibody. All bands in these images co-migrated approximately with a 70 kDa protein standard (PageRuler; ThermoFisher). **(C)** Serum response element (SRE)-luciferase activation by epitope-tagged forms of constitutively active Gα12 and Gα13. Reporter constructs used were SRE-L, encoding firefly luciferase under control of a SRE-containing promoter, and pRL-TK, encoding *Renilla* luciferase governed by a thymidine kinase promoter. HEK293 cells grown in 12-well plates were transfected with SRE-L (0.2 µg) and pRL-TK (0.02 µg), plus plasmids encoding Gα12 or Gα13 as untagged, internally myc-tagged, or internally EGFP-tagged forms (0.1 µg). All constructs harbored activating Gln-to-Leu mutations in the switch II region, Q229L for Gα12 and Q226L for Gα13. Cells were harvested approximately 48 h post-transfection and assayed for luciferase activity as described in Methods. Firefly activity normalized for *Renilla* activity is shown for each sample (Y-axis) and presented as % of the untagged Gα12 or Gα13 response. Data presented are the mean of three independent experiments, with error bars indicating range. **(D)** Binding of Gα13/Gα12 chimeras to regions of RhoGEFs. A schematic of these chimeras, previously reported [47] and provided by Barry Kreutz (Univ. of Illinois at Chicago), is shown with an EGFP tag positioned as described in Methods. Both constructs harbor activating (QL) mutations in the switch II region. Lysates of HEK293 cells transfected with these tagged chimeras were subjected to co-precipitations by RhoGEF domains as described in **(A)** and **(B)**, above. Immunoblot results using anti-EGFP antibody are shown, and are representative of three independent experiments.

**AKAP-Lbc and p114RhoGEF are selective for Gα12 binding.** We next sought to compare Gα12 and Gα13 directly in their ability to engage AKAP-Lbc and p114RhoGEF. Although Gα12 has been linked to AKAP-Lbc-mediated signaling in multiple studies, the role of Gα13 is less clear. Both Gα12 and Gα13 bind the RH domain of RhoGEFs [4], but a role in driving their catalytic activity is better supported for Gα13: stimulation of purified p115RhoGEF by Gα13 but not Gα12 was observed in a reconstituted system measuring RhoA activation, and cell-based studies of p115RhoGEF-mediated Rho signaling also showed selectivity for Gα13 [38,39]. Early studies of AKAP-Lbc suggested a different specificity, with Gα12 showing more potency than Gα13 in assays of AKAP-Lbc mediated RhoA activation in cultured cells [12]. Experiments using dominant-negative Gα12 provided evidence for a Gα12-AKAP-Lbc-Rho axis driving multiple disease states in cardiac myocytes [16,17]; however, similar manipulation of Gα13 signaling was not examined in these studies. Interestingly, Gα13 was implicated in signaling through the AKAP-Lbc splice variants proto-Lbc and Brx [40,41], and a recent report showed Rgnef, a RhoGEF with significant homology to the Gα12-binding region we mapped in AKAP-Lbc, serving as a signaling effector for Gα13 but not Gα12 [42]. In the current study, we developed an approach for side-by-side, single-antibody detection of Gα12 and Gα13 in binding assays by introducing an epitope tag to Gα13 at the same structural position as our recombinant Gα12 constructs. Our original approach for Gα12 was to duplicate its native Pro-139, Val-140 motif within the αB–αC loop and insert a myc epitope tag between these motifs, a strategy that preserved effector binding and Rho-mediated signaling by Gα12 [25,27,43]. To tag Gα13 in our current study, we first engineered a M136P substitution to generate a Pro-Val sequence in the αB–αC loop of Gα13, and then duplicated this motif with a myc tag placed in the same position as our Gα12 constructs. Our initial results using anti-myc antibodies gave a relatively weak immunoblot signal, despite anti-Gα12 and anti-Gα13 antibodies strongly detecting individual tagged proteins (data not shown). Therefore, we replaced the myc tag with enhanced green fluorescent protein (EGFP) in Gα12 and Gα13, and using an anti-EGFP antibody were able to detect both proteins strongly at the predicted MW of ~70 kDa in lysates of HEK293 cells. Expression levels for these recombinant α subunits were similar as determined by anti-EGFP immunoblots (Figure 4A). In co-precipitation experiments tracking constitutively active Gα12 and Gα13 using this single antibody, we observed striking differences in their binding to the 257-residue regions of AKAP-Lbc and p114RhoGEF. Whereas Gα12 showed similar affinity for RH- and non-RH RhoGEFs, Gα13 showed no binding to the AKAP-Lbc or p114RhoGEF domains while exhibiting much stronger interaction with the LARG RH domain than Gα12 (Figure 4A, B). These results suggest AKAP-Lbc and p114RhoGEF share structural features that confer specific interaction with Gα12 within the G12/13 class.

To verify these EGFP-tagged G12/13 α subunits as retaining signaling function through Rho, we examined their stimulation of serum response element (SRE) mediated transcription, a well-characterized readout of G12/13 signaling that is sensitive to Rho inhibition by the C3 exoenzyme of *Clostridium botulinum* [44,45,46]. EGFP-tagged, constitutively active Gα12 and Gα13 were comparable to their myc-tagged and untagged counterparts in driving this pathway in HEK293 cells (Figure 4C). These findings provided evidence that the EGFP adduct does not disrupt α subunit folding or interfere with G12/13 signaling function.

We next utilized chimeric versions of Gα12 and Gα13 to investigate structural differences between these proteins that underlie preferential binding of Gα12 by AKAP-Lbc and p114RhoGEF. Within these chimeras, a transition occurs from Gα13 to Gα12 immediately upstream or downstream of the switch regions [47]. To compare these chimeras with Gα12 and Gα13 in binding assays, we engineered each protein to harbor an internal EGFP tag as described above. Both chimeras exhibited strong binding to the LARG RH domain but no interaction with the Gα12-binding region of AKAP-Lbc or p114RhoGEF (Figure 4D). Because these binding profiles resemble Gα13 rather than Gα12, we conclude the N-terminal region of Gα12 provides the crucial determinants of its selective binding to AKAP-Lbc and p114RhoGEF. These results further support a mechanism of G protein coupling in these RhoGEFs that differs from classical RH-RhoGEFs, as earlier studies of these Gα12/Gα13 chimeras implicated amino acid determinants C-terminal of the switch regions as conferring specificity for Gα13 vs. Gα12 in driving RH-RhoGEF activity [47].

**Figure 5:**
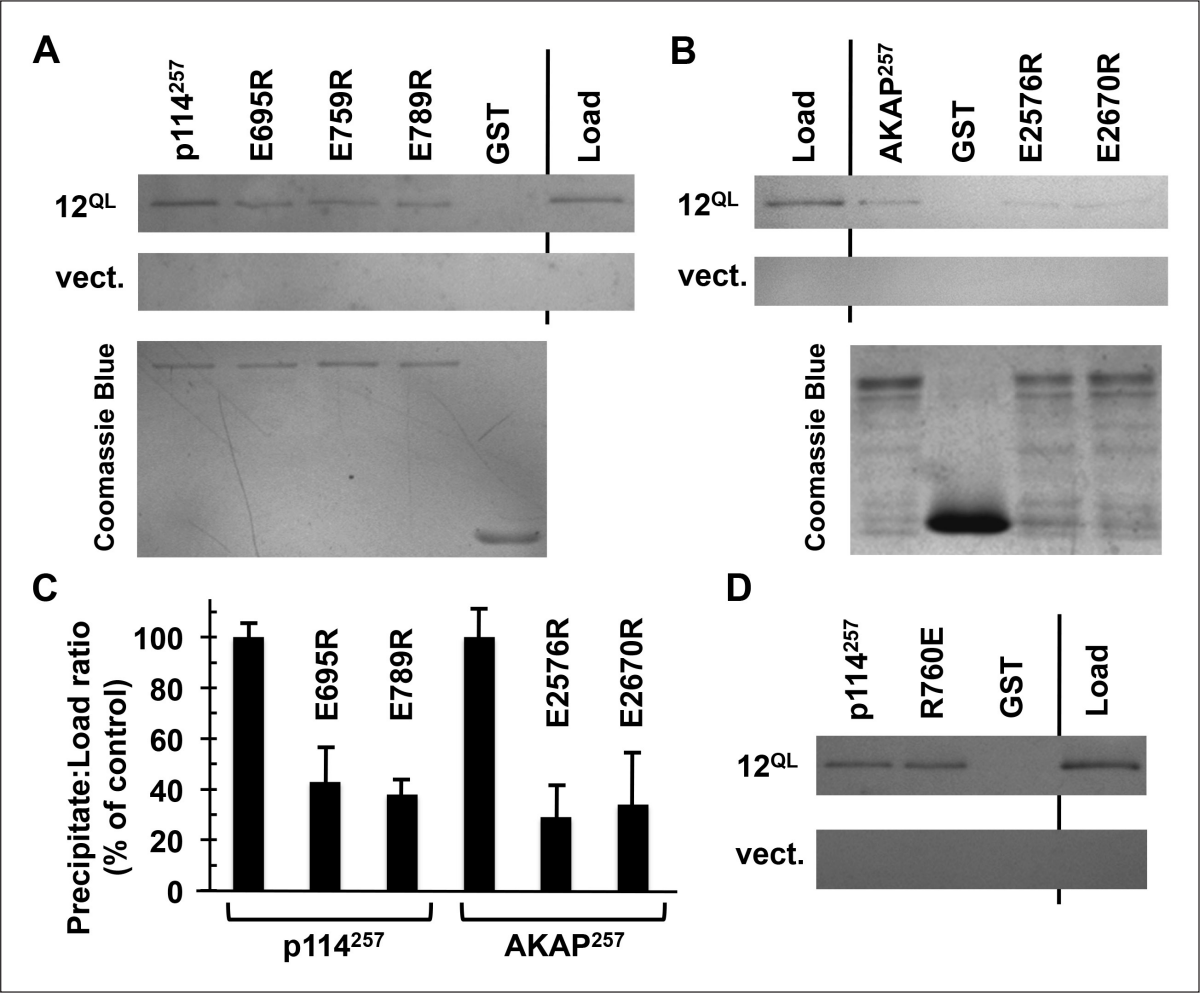
Conserved residues in AKAP-Lbc and p114RhoGEF participate in Gα12 binding. **(A)** Interaction of p114RhoGEF charge-reversal mutants with Gα12. The indicated mutants engineered in our GST-fused 257-amino acid region of p114RhoGEF were expressed in *E. coli* and immobilized on Sepharose. Concentrations of mutant p114RhoGEF proteins were adjusted so that approximately equal concentrations would be compared for ability to co-precipitate constitutively active Gα12 (*12^QL^*). For each sample, 20% of volume was analyzed by Coomassie Blue staining (lower image) to confirm uniform levels of immobilized p114RhoGEF variants. **(B)** AKAP-Lbc charge-reversal mutants were examined for Gα12 co-precipitation using the same procedure described for p114RhoGEF mutants. **(C)** For co-precipitations of Gα12, bands were quantified using ImageJ. Precipitate:load values for each mutant were calculated and presented as % of this value for non-mutated p114RhoGEF or AKAP-Lbc regions, which were set at 100%. Graphical data represent two or more independent experiments, with mean ± range shown. **(D)** Co-precipitation of Gα12 by an immobilized 257-residue p114RhoGEF construct harboring a charge-reversal of the Rgnef-homologous Arg residue (*R760E*). Images are representative of two independent experiments.

**Conserved residues in AKAP-Lbc and p114RhoGEF participate in Gα12 binding.** Within the regions of AKAP-Lbc and p114RhoGEF we identified as sufficient for binding Gα12, we sought to identify shared residues important for Gα12 interaction. In the 106-amino acid domain of p114RhoGEF with close homology to AKAP-Lbc, several positions were noted as sharing an identical charged residue. We engineered charge-reversals at these positions in the encompassing 257 residues of p114RhoGEF, and assessed Gα12 binding by these GST-fusion proteins. These experiments identified three Glu residues potentially involved in Gα12 binding, as evidenced by Glu-to-Arg mutations at these positions causing diminished co-precipitation of constitutively active, myc-tagged Gα12 (Figure 5A, C). For these mutants of p114RhoGEF- E695R, E759R, and E789R- we engineered charge substitutions at the corresponding positions in AKAP-Lbc and found two variants (E2576R and E2670R) partially disrupted in Gα12 interaction (Figure 5B, C). These results identify charged amino acids shared between AKAP-Lbc and p114RhoGEF that may contribute to a conserved Gα12-interacting surface, and suggest these non-RH RhoGEFs utilize similar structural mechanisms in receiving communication from activated Gα12.

During preparation of our manuscript, Aragay and colleagues reported an interaction between Gα13 and Rgnef [42], a RhoGEF lacking an RH domain. Interestingly, Rgnef was found not to bind Gα12 in this recent article. In comparison to the 106-residue regions with 47% identity in AKAP-Lbc and p114RhoGEF, this Rgnef region has slightly lower homology and lacks Glu residues at the positions we identified in AKAP-Lbc and p114RhoGEF as involved in Gα12 interaction (Figure 5A, B). Mutation of a key Arg residue of Rgnef was shown by Masia-Balague et al. to disrupt interaction with Gα13 [42], and this Arg is located in the 106-residue regions of close homology in AKAP-Lbc and p114RhoGEF. Because p114RhoGEF shows closest similarity to Rgnef in this region (42% identity) we engineered a charge-reversal at this position, Arg-760, in our GST-fusion of p114RhoGEF. As shown in Figure 5D, this R760E mutation did not impair p114RhoGEF interaction with constitutively active Gα12. Together with the report of Gα13-Rgnef interaction, our results suggest this region of homology in AKAP-Lbc, p114RhoGEF and Rgnef is derived from a G12/13 binding motif in an ancestral RhoGEF, but became specialized for Gα13 binding in Rgnef and Gα12 binding in a more recent common ancestor of AKAP-Lbc and p114RhoGEF.

**Figure 6:**
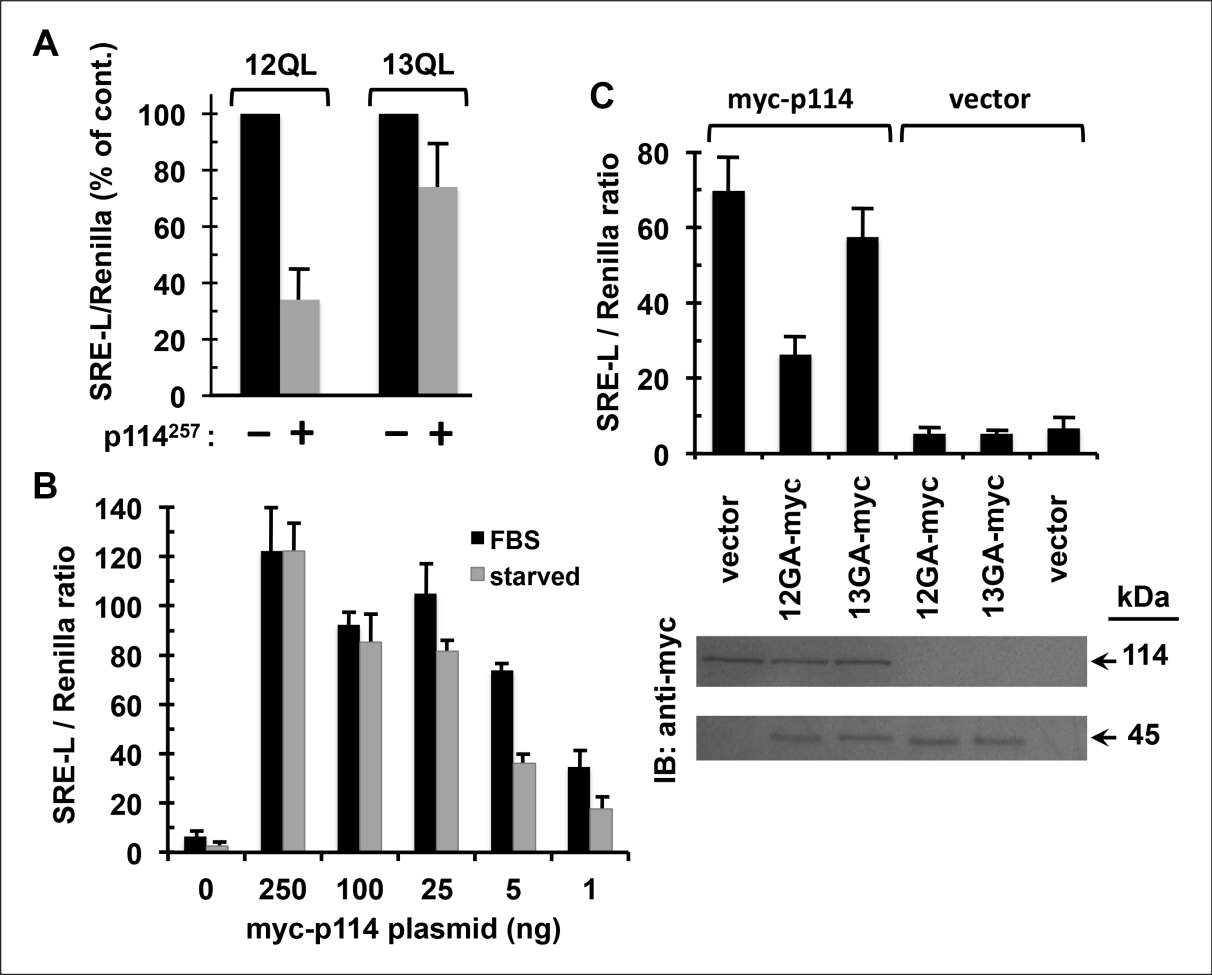
Preferential coupling of p114RhoGEF to Gα12 in signaling to SRE. **(A)** Effects of an ectopically expressed region of p114RhoGEF on Gα12- and Gα13-mediated signaling. HEK293 cells grown in 12-well plates were transfected with myc-tagged, constitutively active Gα12 or Gα13, plus either a construct encoding the Gα12-interacting 257 amino acids of p114RhoGEF with an N-terminal FLAG-tag (*p114^257^*) or empty vector. All cells were co-transfected with the firefly luciferase reporter SRE-L (0.2 µg) and the *Renilla* luciferase reporter pRL-TK (0.02 µg). Approximately 48 h post-transfection, cells were harvested and assayed for firefly and *Renilla* luciferase activity as described in Methods. For Gα12 and Gα13 transfections, effects of the p114RhoGEF construct are presented as % of vector control results, which were set at 100% for each α subunit. Graphs indicate mean ± s.e.m. for three independent experiments. **(B)** Effects of p114RhoGEF titration on serum dependence of its signaling to SRE. HEK293 cells grown in 12-well plates were transfected in duplicate with decreasing amounts of plasmid encoding myc-tagged, full-length p114RhoGEF (X-axis), plus uniform amounts of SRE-L and pRL-TK. After 32 h, one sample per transfection condition was washed twice with DMEM and serum-starved in the same medium for 12 h, whereas the duplicate sample received washes and addition of DMEM containing 10% FBS. Cell lysates were assayed for firefly and *Renilla* luciferase activity. Data shown are the mean of two independent experiments, with error bars indicating range. **(C)** Effects of dominant-negative Gα12 and Gα13 on serum-dependent signaling through p114RhoGEF. HEK293 cells were transfected with 5 ng plasmid encoding myc-tagged, full-length p114RhoGEF (*myc-p114*) or 5 ng empty vector, along with 50 ng plasmid encoding myc-tagged, constitutively GDP-bound variants of Gα12 (*12GA-myc*) or Gα13 (*13GA-myc*). All cells were co-transfected with SRE-L and pRL-TK and grown in DMEM + 10% serum for approximately 48 h, then assayed by luminometry. Data presented are mean ± s.e.m. for three independent experiments. At bottom are results of SDS-PAGE/immunoblot analysis of cell lysates, with expression of myc-p114RhoGEF (upper panel) and myc-tagged, dominant-negative G12/13 α subunits (lower panel) tracked using an anti-myc antibody. Immunoblots shown are a representative of three independent experiments.

**Preferential coupling of p114RhoGEF to Gα12 in signaling to SRE.** Because interaction between Gα12 and AKAP-Lbc is well-described as driving Rho activation and a variety of downstream cellular events, we sought evidence of Gα12-p114RhoGEF interaction playing a signal transduction role in cells. The p114RhoGEF region we defined as Gα12-binding lacks the tandem DH/PH domains required for Rho activation. Therefore, we predicted this isolated polypeptide could occupy the activated α subunit and disrupt signaling through native p114RhoGEF, which is a protein expressed in HEK293 cells [33]. For a signaling readout we utilized SRE-mediated transcriptional activation, which is well-characterized as a growth signaling response stimulated by Gα12 or Gα13 activation [48]. This assay utilized a reporter plasmid in which expression of firefly luciferase is governed by SRE, a promoter element of the *c-fos* proto-oncogene bound by serum response factor in the nucleus [49]. We engineered the Gα12-interacting, 257 amino acid region of p114RhoGEF as a FLAG epitope-tagged construct and transfected this into HEK293 cells along with the SRE-luciferase (SRE-L) reporter. Expression of this p114RhoGEF construct was more disruptive to signaling by constitutively active Gα12 than Gα13 (Figure 6A), providing evidence this region of p114RhoGEF can bind Gα12 selectively in a cellular context and interfere with its signaling in a Rho-mediated pathway.

To further examine coupling of the G12/13 subfamily to p114RhoGEF in cells, we tested whether specific disruption of Gα12 or Gα13 signaling in cells would affect a receptor-driven response involving p114RhoGEF. In agreement with earlier studies [33], expression of myc-tagged, full-length p114RhoGEF robustly stimulated SRE-mediated transcription in HEK293 cells (Figure 6B). In our initial transfections, we observed no decrease in SRE signaling when serum was removed from the culture media. However, after titrating the amount of myc-p114RhoGEF plasmid downward in these transfections, we observed a range of 1–5 ng DNA in which the presence of serum caused more than a two-fold increase in p114RhoGEF-dependent SRE activation (Figure 6B). Therefore, we transfected cells with 5 ng of myc-p114RhoGEF plasmid along with constructs encoding myc-tagged, constitutively GDP-bound mutants of Gα12 or Gα13. This dominant-negative Gα12 (G228A mutation) was disruptive to p114RhoGEF-mediated signaling, causing a 66% drop in the serum effect on SRE-mediated transcription, whereas dominant-negative Gα13 (G225A mutation) had a much smaller effect (Figure 6C). Immunoblot analysis confirmed uniform expression of these dominant-negative, myc-tagged G12/13 α subunits as well as myc-p114RhoGEF. These results suggest endogenous Gα12 participates in receptor-driven signaling through p114RhoGEF.

## Discussion

A key question regarding the Gα12-AKAP-Lbc-Rho signaling axis is: what is the mechanism utilized by Gα12 to bind and activate AKAP-Lbc? Gα12 stimulation of AKAP-Lbc is implicated in heart development as well as pathologies that include cardiac hypertrophy and pro-fibrotic differentiation [14,16,17]. To develop therapies for manipulating these pathways, it will be valuable to understand the fine structural features of Gα12-AKAP-Lbc interaction. RH (RGS-homology) RhoGEFs are well characterized effectors of the G12/13 subfamily for driving Rho activation, and the RH domain is the primary component of interaction with the activated α subunit [4]. However, the lack of an RH domain in AKAP-Lbc evokes the question of how Gα12 binds this protein and stimulates its RhoGEF activity. A mechanism was suggested by surface plasmon resonance studies that showed the tandem DH/PH domains of LARG providing a Gα13-binding surface, along with more recent structural evidence that Gα13 forms a contact with the DH domain of p115RhoGEF to stimulate its activity [50,51]. However, early studies of proto-Lbc cast doubt on its DH/PH domains participating in Gα12 interaction, as a proto-Lbc construct lacking these domains retained Gα12 binding and exhibited dominant-negative effects on Gα12-mediated cell rounding [29,52]. In our current study, we aligned AKAP-Lbc sequence outside its DH/PH domains with proteins that bind selectively to Gα12, and identified a AKAP-Lbc region that bound constitutively active Gα12 when expressed as a GST-fusion. We further identified a homologous region in another RhoGEF lacking an RH domain, p114RhoGEF, and showed this region to bind Gα12, revealing p114RhoGEF as a novel Gα12 target. Within these Gα12-interacting regions of AKAP-Lbc and p114RhoGEF, close homology is limited to a 106-residue subregion in which our mutational studies identified two Glu residues involved in Gα12 binding. However, GST-fusions of these isolated subregions did not bind Gα12, whereas inclusion of flanking sequence to yield a 257-amino acid region allowed Gα12 interaction (see Figure 2). Although it is possible that amino acids in AKAP-Lbc and p114RhoGEF flanking the “core” sequence provide determinants of Gα12 binding, lack of homology between AKAP-Lbc and p114RhoGEF in these flanking regions suggests they are more likely needed to facilitate correct folding of the core subregion. A similar requirement has been shown for the RGS box of p115RhoGEF, which requires an adjacent 60-amino acid region for correct folding and stability [21]. Gains in structural understanding of AKAP-Lbc and p114RhoGEF outside their tandem DH/PH domains should shed light on the folding of this conserved 106-residue region and the role of contextual amino acids.

AKAP-Lbc and p114RhoGEF lack the RH domain that is characteristic of the classical G12/13-responsive RhoGEFs, yet are grouped with these proteins in the Lbc subclass of RhoGEFs based on sequence homology [3]. Because this small group shares the unique property of G12/13 responsiveness among the 70 RhoGEFs that harbor DH domains [53], it is tempting to speculate that the Gα12-binding region we mapped in AKAP-Lbc and p114RhoGEF generates a fold that functions in similar fashion to an RH domain. A previous report described a C-terminal region of proto-Lbc as similar to a consensus Lsc-homology (LH) region derived from LARG, p115RhoGEF, and PDZ-RhoGEF, in which this LH region mostly encompasses the RH domain [29]. However, the 106-residue region of interest in AKAP-Lbc overlaps only partially with this putative LH region in proto-Lbc, and among the 50 amino acids identical between AKAP-Lbc and p114RhoGEF in this region (see Figure 2A), only six matched the consensus LH sequence from RH-RhoGEFs. Therefore, we believe it is unlikely these regions of AKAP-Lbc and p114RhoGEF provide a RH-like surface. This conclusion is supported by our experiments in which a Cys-11 mutation hindered Gα12 binding to these regions without disrupting its interaction with the RH domain of LARG, along with our finding that a Gα12 cassette mutant uncoupled from RH-RhoGEFs showed normal binding to AKAP-Lbc and p114RhoGEF. We propose these sequences in AKAP-Lbc and p114RhoGEF define a novel Gα12-binding domain unique to these non-RH RhoGEFs, so that coupling between Gα12 and these proteins utilizes a signaling mechanism that is fundamentally different from the well-studied mechanism in which Gα13 engages canonical RH-RhoGEFs.

By installing a common epitope tag in the helical domain of Gα12 and Gα13, we were able to perform comparative binding assays in which G12/13 α subunits were detected by a single antibody in co-precipitates of immobilized RhoGEF domains. To our knowledge, this is the first time this approach has been used for direct comparisons of Gα12 and Gα13 binding to specific effector proteins. AKAP-Lbc and p114RhoGEF interacted with Gα12 but not Gα13 in this side-by-side assay, and our follow-up experiments in cultured HEK293 cells showed the isolated p114RhoGEF region exerting dominant-negative effects preferentially on Gα12-mediated growth signaling in comparison to Gα13. Furthermore, a dominant-negative Gα12 showed greater potency than its Gα13 counterpart in disrupting serum-mediated signaling through overexpressed p114RhoGEF in these cells. An inference from these findings is that some G protein-RhoGEF pairings evolved to utilize Gα13 for signaling input while others were honed during evolution to employ Gα12. Early studies of purified RH-RhoGEFs showed preferential coupling to Gα13: the RhoGEF activity of p115RhoGEF and LARG was stimulated in a reconstituted system by GTP-bound Gα13, whereas GTP-bound Gα12 failed to stimulate p115RhoGEF under these conditions and stimulated LARG only if the latter protein was previously phosphorylated by Tec kinase [38,54]. Also, p115RhoGEF acted synergistically with Gα13 but not Gα12 in stimulating SRE-mediated transcription in cells [39]. Gα12 and Gα13 arose from a gene duplication event prior to the divergence of lampreys and gnathostomes, and all invertebrates encode a single G12/13 α subunit [55]. RH-RhoGEFs are found in *Drosophila* and *Caenorhabditis elegans*, where they participate in G12/13 signaling to Rho that drives cell shape changes and other responses [56,57]. However, RhoGEFs harboring regions of homology to the Gα12-binding domains of AKAP-Lbc and p114RhoGEF are not found in these organisms. Therefore, we propose the domain shared between AKAP-Lbc and p114RhoGEF represents a recently evolved signaling entryway for Gα12 to regulate these non-RH RhoGEFs, in contrast to the RH domain that represents an ancient structural feature that appears to have become specialized for Gα13 coupling during vertebrate evolution. The finding that LARG acquires responsiveness to Gα12 if previously phosphorylated by Tec kinase [54] evokes the interesting possibility that other G12/13 responsive RhoGEFs, including AKAP-Lbc and p114RhoGEF, could harbor structural features that allow modulation of their coupling to a specific G12/13 input.

An additional RhoGEF in the Lbc subclass, Rgnef (p190RhoGEF), harbors a 106-residue region similar to the Gα12-binding regions of AKAP-Lbc and p114RhoGEF but with slightly lower homology. In sharp contrast to the preferential Gα12 binding we observed for AKAP-Lbc and p114RhoGEF, Rgnef shows functional interaction with Gα13 but not Gα12 [42]. These findings suggest a G12/13-responsive domain existed in a common ancestor of AKAP-Lbc, p114RhoGEF, and Rgnef, and after the gene duplication event that produced Gα12 and Gα13, the Rgnef lineage evolved selectivity for Gα13 while the lineage that yielded AKAP-Lbc and p114RhoGEF was tuned to become specialized for Gα12 interaction. Alignment of Rgnef with the Gα12-interacting regions in Figure 2A supports such a model. For example, AKAP-Lbc harbors a motif (EQEKQRSLEKQR) almost identical to p114RhoGEF (EQERQRNFEKQR) yet Rgnef shows much lower identity at these positions (QDQKSRDADRQH). However, other spans are highly similar to Rgnef, and overall the Rgnef sequence shows 42% identity with p114RhoGEF and 35% identity with AKAP-Lbc in these 106-residue regions. Perhaps importantly, the two Glu residues we mapped in AKAP-Lbc and p114RhoGEF as participating in Gα12 interaction are absent from the corresponding positions in Rgnef. An interesting area of future work will be to interchange amino acids within these RhoGEFs to elucidate their respective mechanisms of Gα12 or Gα13 specificity.

Our discovery that p114RhoGEF harbors a Gα12-specific binding region provides the first evidence of a signaling link between p114RhoGEF and a G protein β subunit. Previous studies of p114RhoGEF suggested multiple binding sites for the G protein β_1_/γ_2_ dimer, and deletion of its DH/PH domains disrupted β_1_/γ_2_-mediated stimulation of its activity, implicating these tandem domains as a β_1_/γ_2_-interacting surface [58]. In addition, we observed constitutively GDP-bound Gα12 selectively interfering with serum-induced signaling through p114RhoGEF that culminates in SRE-mediated transcriptional activation. Taken together, our findings suggest the presence of a signaling pathway in which Gα12 couples to p114RhoGEF. In contrast to the mostly heart-specific expression of AKAP-Lbc, p114RhoGEF is expressed in numerous tissues and cell lines [12,33]. Several studies in recent years have revealed roles for this RhoGEF in physiologic and pathologic events that include tight junction assembly, establishment of epithelial polarity in retinal development, and myosin phosphorylation in tumor cell migration [59,60,61,62]. Also, studies in three-dimensional cell culture revealed a role for p114RhoGEF in epithelial tubule formation [63]. The ubiquitous expression patterns of Gα12 and p114RhoGEF provide a variety of cell types in which interaction between these proteins could play signaling roles.

## Conclusion

Many studies have revealed key biochemical and structural details of G12/13 signaling through the RH-RhoGEFs LARG, p115RhoGEF, and PDZ-RhoGEF. However, the mechanisms utilized by this heterotrimeric G protein subfamily in stimulating non-canonical effectors such as AKAP-Lbc are poorly understood. Our results identify a Gα12-binding region within AKAP-Lbc, and provide the first evidence that p114RhoGEF interacts with Gα12 through a region highly similar to this AKAP-Lbc sequence. This region of AKAP-Lbc and p114RhoGEF is specific for Gα12 binding, in contrast to the preferential Gα13 coupling observed for RH-RhoGEFs. These results define a novel mechanism for Gα12 input to a RhoGEF-Rho pathway distinct from the well-characterized signaling axis in which RH-RhoGEFs connect the G12/13 subfamily to Rho. Absence of invertebrate RhoGEFs with homology to this Gα12-binding domain of AKAP-Lbc and p114RhoGEF suggests recent evolution of this structural feature, in contrast to the ancient RH domains conserved in G12/13-responsive RH-RhoGEFs.

## Abbreviations

A-kinase anchoring protein (AKAP)

DH, Dbl-homology

EGFP, enhanced green fluorescent protein

G protein, trimeric guanine nucleotide binding protein

GST, glutathione-S-transferase

LARG, leukemia-associated RhoGEF

PH, pleckstrin-homology

RGS, regulator of G protein signaling

RH, RGS-homology

RhoGEF, Rho-directed guanine nucleotide exchange factor

s.e.m., standard error of the mean

SRE, serum response element
